# The Complete Genome Sequence of ‘*Candidatus* Liberibacter solanacearum’, the Bacterium Associated with Potato Zebra Chip Disease

**DOI:** 10.1371/journal.pone.0019135

**Published:** 2011-04-28

**Authors:** Hong Lin, Binghai Lou, Jonathan M. Glynn, Harshavardhan Doddapaneni, Edwin L. Civerolo, Chuanwu Chen, Yongping Duan, Lijuan Zhou, Cheryl M. Vahling

**Affiliations:** 1 United States Department of Agriculture-Agricultural Research Service, CDPG, San Joaquin Valley Agricultural Sciences Center, Parlier, California, United States of America; 2 Guangxi Citrus Research Institute, Guilin, Guangxi, People's Republic of China; 3 Carver Center for Genomics, Department of Biology, University of Iowa, Iowa City, Iowa, United States of America; 4 United States Department of Agriculture-Agricultural Research Service, USHRL, Fort Pierce, Florida, United States of America; 5 Department of Plant Pathology, University of Florida, Gainesville, Florida, United States of America; University of Wisconsin-Milwaukee, United States of America

## Abstract

Zebra Chip (ZC) is an emerging plant disease that causes aboveground decline of potato shoots and generally results in unusable tubers. This disease has led to multi-million dollar losses for growers in the central and western United States over the past decade and impacts the livelihood of potato farmers in Mexico and New Zealand. ZC is associated with ‘*Candidatus* Liberibacter solanacearum’, a fastidious alpha-proteobacterium that is transmitted by a phloem-feeding psyllid vector, *Bactericera cockerelli* Sulc. Research on this disease has been hampered by a lack of robust culture methods and paucity of genome sequence information for ‘*Ca.* L. solanacearum’. Here we present the sequence of the 1.26 Mbp metagenome of ‘*Ca.* L. solanacearum’, based on DNA isolated from potato psyllids. The coding inventory of the ‘*Ca.* L. solanacearum’ genome was analyzed and compared to related *Rhizobiaceae* to better understand ‘*Ca.* L. solanacearum’ physiology and identify potential targets to develop improved treatment strategies. This analysis revealed a number of unique transporters and pathways, all potentially contributing to ZC pathogenesis. Some of these factors may have been acquired through horizontal gene transfer. Taxonomically, ‘*Ca.* L. solanacearum’ is related to ‘*Ca.* L. asiaticus’, a suspected causative agent of citrus huanglongbing, yet many genome rearrangements and several gene gains/losses are evident when comparing these two Liberibacter. species. Relative to ‘*Ca.* L. asiaticus’, ‘*Ca.* L. solanacearum’ probably has reduced capacity for nucleic acid modification, increased amino acid and vitamin biosynthesis functionalities, and gained a high-affinity iron transport system characteristic of several pathogenic microbes.

## Introduction

Zebra chip (ZC) is an economically important disease of potato (*Solanum tuberosum*). The disease has been reported since the early 1990s in Central America and Mexico, and was found in the United States in 2000. The disease reduces the marketability of potatoes because it causes discoloration of the medullary rays in raw tubers and intensely dark discoloration when tubers are processed into chips. Tubers from ZC-affected plants also have poor germination rates [1]. The etiology of ZC has not been conclusively determined, although the disease is identified to be associated with a fastidious alpha-proteobacterium named “*Candidatus* Liberibacter solanacearum’ [2]. The disease is also associated with the potato psyllid, *Bactericera cockerelli*, which harbors ‘*Ca.* L. solanacearum’ as part of its gut microflora and is thought to transmit the pathogen while feeding on host phloem sap [3]. ‘*Ca.* L. solanacearum’ is also associated with diseases of other solanaceous crops in New Zealand [2] and carrot yellows in Finland [4].

‘*Ca.* L. solanacearum’ is not the only Liberibacter species associated with plant diseases. Three other phylogenetically-distinct [5] species of Liberibacter are associated with citrus Huanglongbing (HLB) [6]. The genome of one of these, “*Candidatus* Liberibacter asiaticus’, has been sequenced and annotated [7]. Because the Liberibacter species associated with ZC and HLB are unculturable, detailed information regarding their etiology, general physiology, and mode of pathogenesis is lacking. To gain further insights into the biology of this genus of bacteria and determine how they contribute to plant decline, we aimed to obtain the complete genome sequence of ‘*Ca.* L. solanacearum’ using metagenomics. Here we present the complete genome sequence of ‘*Ca.* L. solanacearum’, identifying several chromosomal features and making predictions about its physiology based on its gene inventory. In addition, we performed comparative analysis between ‘*Ca.* L. solanacearum’ and ‘*Ca.* L. asiaticus’ to better understand how these microbes cause diseases in plants. The results provide genomic data supporting a high degree of similarity between ZC-associated ‘*Ca.* L. solanacearum’ and HLB-associated ‘*Ca.* L. asiaticus’, congruent on their similar lifestyles as phloem-colonizing psyllid-vectored bacteria [2], [3], [7], [8], [9]. However, we found several significant differences between these closely-related species with regard to their genome organization, biosynthetic capacity for vitamins and amino acids, potential for nucleic acid modification and restriction, and nutrient uptake systems. These unique attributes are likely related to their lifestyle and host range. The data presented here offer critical insights into the physiology of the ‘*Ca.* L. species that could facilitate development of novel treatment strategies for both ZC and HLB.

## Results and Discussion

### ‘*Candidatus* Liberibacter solanacearum’ sequence generation and assembly

Two rounds of 454 pyrosequencing were carried out to obtain the complete ‘*Ca.* L. solanacearum’ genome sequence (GenBank accession # CP002371). The initial round of sequencing was done using the FLX standard pyrosequencing method [10]. This run generated a total of 176,935 reads yielding 36,831,668 base pairs (bp) with an average read length of 208 bp. These reads underwent *de novo* assembly into 15,061 contigs covering 5,535,163 bp with contig lengths ranging from 500–55,601 bp. From this dataset, 134 contigs were identified based on homology searches and subsequently confirmed to be valid ‘*Ca.* L. solanacearum’ sequences by PCR. The second round of sequencing was conducted by Titanium pyrosequencing [10]. This run generated 513,784 reads with a total of 208,868,707 base pairs with average read length of 406 bp. The total sequencing reads from the second round of sequencing were then used for *de novo* assembly, generating 18,147 contigs covering 9,768,772 bp. Of these, 27 contigs ranging from 1,000–279,292 bp were identified as homologous to known Liberibacter genomic DNA sequences, and these were subsequently confirmed by PCR. Together, both rounds of DNA sequencing generated a composite sequence dataset with at least 30 fold coverage of the ‘*Ca.* L. solanacearum’ genome.

**Figure 1:**
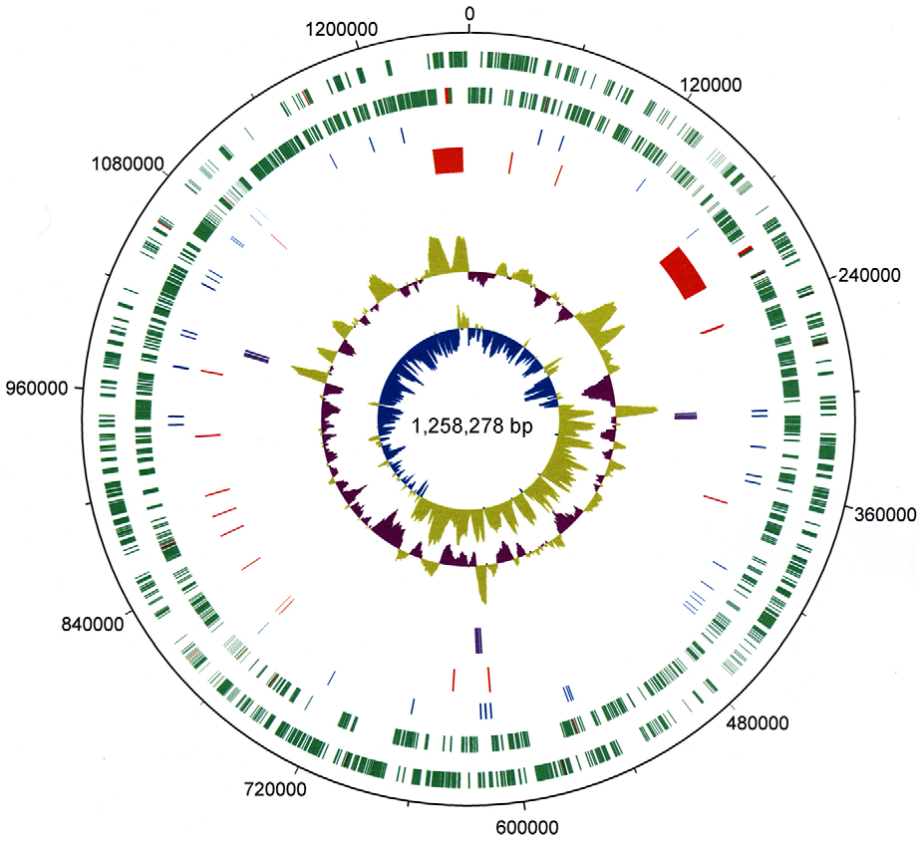
Schematic representation of the *‘Candidatus* Liberibacter solanacearum’ genome. Circular representation of the 1.26 Mbp genome. The tracks from the outmost circles represent (1) Forward CDS (green) and (2) Reverse CDS (green) with pseudogenes in red; (3) tRNA (blue); (4) bacteriophage derived regions and probable phage remnants (red); (5) three copies of rRNA operon (16S, 23S and 5S) (purple); (6) % G+C content and (7) GC skew [(GC/(G+C))].

To confirm and connect ‘*Ca.* L. solanacearum’ contigs, 350 primer pairs were designed and used for conventional and long distance PCR (Table S1). Using method we developed, 136 primers were designed for genomic walking [11] (Table S2). Amplicons generated from these primers were directly sequenced or cloned prior to sequencing. In total, we resequenced over 200,000 bp by Sanger sequencing. These efforts led to the successful closure of all gaps in the genome sequence and resulted in assembly of a circular chromosome consisting of 1,258,278 bp (Figure 1).

### General features of the ‘*Candidatus* Liberibacter solanacearum’ genome and comparison to ‘*Ca.* L. asiaticus’

**Figure 2:**
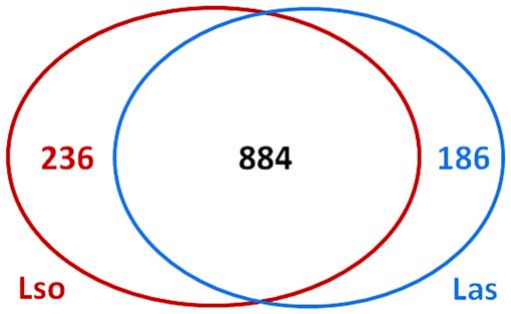
Comparison of the ‘*Candidatus* Liberibacter solanacearum’ and ‘*Candidatus* Liberibacter asiaticus’ predicted proteomes. ‘*Ca.* L. solanacearum’-encoded sequences are delimited by the red line and ‘*Ca.* L. asiaticus’-encoded sequences are delimited by the blue line. 884 bidirectional best hits (BBH) were identified in both genomes using an e-value cutoff of 10^−15^. The remaining sequences consist of both unidirectional hits and unique sequences. Using the above cutoff values, 236 BBH ‘*Ca.* L. solanacearum’ sequences are considered unique to ‘*Ca.* L. solanacearum’ and 186 BBH ‘*Ca.* L. asiaticus’ sequences are considered unique to ‘*Ca.* L. asiaticus’. Most of the species-specific predicted translations are annotated as hypothetical proteins.

The ‘*Ca.* L. solanacearum’ genome has 35.24% G+C content (Table 1), which is considerably lower than the ∼60% G+C content observed for most other genomes of the *Rhizobiaceae* [12], [13], [14], but similar to the G+C content of the ‘*Ca.* L. asiaticus’ genome (36.48%) [7]. The 1.26 Mbp ‘*Ca.* L. solanacearum’ chromosome encodes 1,192 putative proteins (CDS); 848 of these can be assigned to a Cluster of Orthologous Groups (COG) and approximately 35% of the total coding sequences encode hypothetical proteins (Table 1). We also identified 3 complete rRNA operons (16S, 23S, and 5S), 45 genes encoding tRNAs, and at least 35 probable pseudogenes within the ‘*Ca.* L. solanacearum’ genome (Table 1). Although the genome size and number of genes encoded by ‘*Ca.* L. solanacearum’ are smaller than most members of the *Rhizobiaceae* family [12], [13], [14], these characteristics are consistent with the general features of the ‘*Ca.* L. asiaticus’ genome [7]. A pairwise comparison of the ‘*Ca.* L. solanacearum’ and ‘*Ca.* L. asiaticus’ genomes revealed 884 protein-coding sequences common to both organisms (Figure 2 and Table S3). Notably, 236 sequences from ‘*Ca.* L. solanacearum’ have no corresponding ortholog in ‘*Ca.* L. asiaticus’ and nearly 90% of these unique sequences encode hypothetical proteins (Figure 2 and Table S4). Conversely, 186 sequences from ‘*Ca.* L. asiaticus’ have no corresponding ortholog in ‘*Ca.* L. solanacearum’ and approximately 95% of these encode hypothetical proteins (Figure 2 and Table S4). Because both ‘*Ca.* L. solanacearum’ and ‘*Ca.* L. asiaticus’ encode for a large number of membrane transporters, we also compared the general transporter capabilities of the two bacteria. Based on our analysis, ‘*Ca.* L. solanacearum’ harbors only 8 additional proteins involved in transport (Table S5). Other genes that show similarity to previously characterized proteins imperative for proper cell function are also discussed below.

**Table 1:** General features of the ‘*Candidatus* Liberibacter solanacearum’ genome.

Feature	Value
Size (bp)	1,258,278
G+C Content	35.24%
CDS (Protein-coding genes)	1,192
Hypothetical Proteins	405
tRNA genes	45
rRNA Operons	3
Putative pseudogenes (frameshifted ORFs)	35

### Organization of the ‘*Candidatus* Liberibacter solanacearum’ genome and identification of prophage-like regions

**Figure 3:**
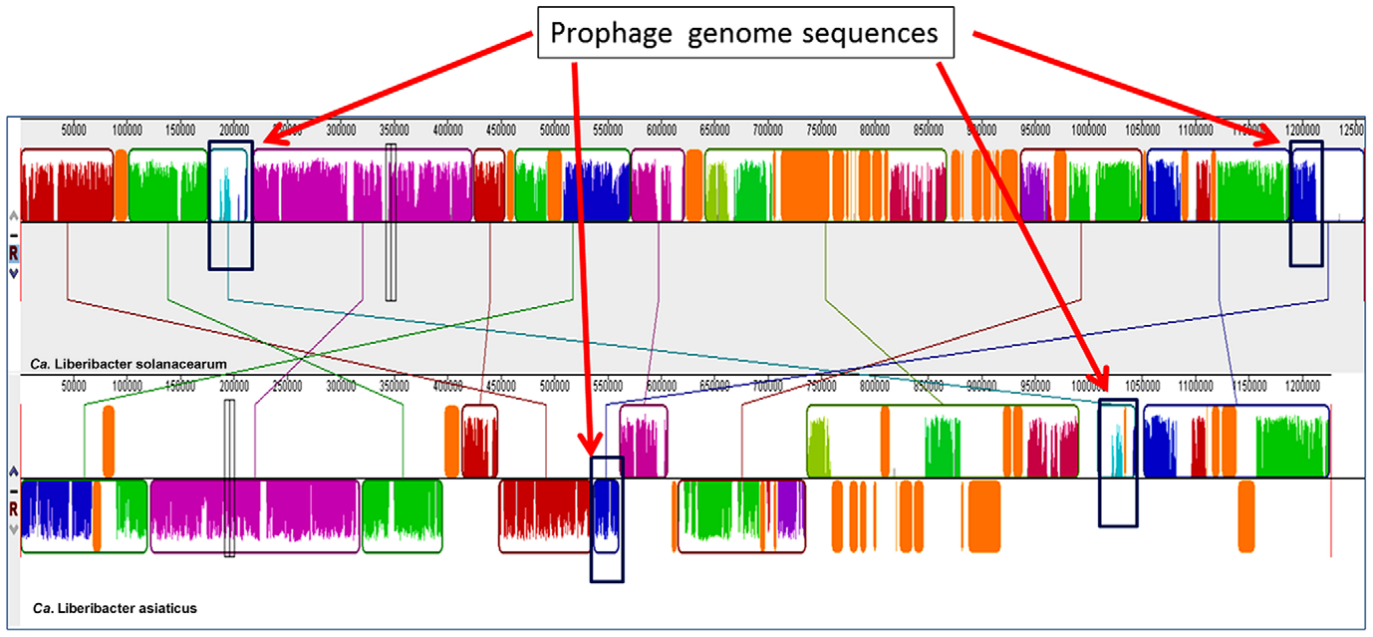
Comparison of the ‘*Candidatus* Liberibacter asiaticus’ and ‘*Candidatus* Liberibacter asiaticus’ chromosomal features. Locally collinear blocks (LCBs) identified genomes of ‘*Ca.* L. solanacearum’ and ‘*Ca.* L. asiaticus’. Each contiguously colored region is a LCB, a region without rearrangement of homologous backbone sequence. LCBs below a genome's center line are in the reverse complement orientation relative to the reference genome, ‘*Ca.* L. solanacearum’. Lines between genomes trace each orthologous LCB through every genome. The ‘*Ca.* L. solanacearum’ and ‘*Ca.* L. asiaticus’ genomes have undergone considerable genome rearrangements. Two rectangles represent bacteriophage-derived regions in ‘*Ca.* L. solanacearum’ which was matched with ‘*Ca.* L. asiaticus’.

While ‘*Ca.* L. solanacearum’ and ‘*Ca.* L. asiaticus’ are phylogenetically related based on 16S rRNA comparisons [2], [3], [5], [15], the organization of these two genomes is different. Alignment of the two genomes suggests several recombination events have occurred since the divergence of these two species from a common ancestor (Figure 3). The identification of two highly-similar ∼40 kb segments within the ‘*Ca.* L. solanacearum’ genome that appear to be phage-derived suggest that phage integration events may be playing a key role in the rearrangement of the Liberibacter genomes (Figure 3 and Figure S1). The first segment, Prophage I (P-I) located from base pair 176,396 to 217,189 in the ‘*Ca.* L. solanacearum’ genome while the second segment, Prophage II (P-II), extends from base pair 1,214,970–1,258,278 (Figure 3). Alignment analysis revealed that P-I had a high degree of similarity with one of the ‘*Ca.* L. asiaticus’ phage sequences whereas the P-II sequence only contained a small segment with a lower degree of similarity to the ‘*Ca.* L. asiaticus’ phage sequences. Several lines of evidence exist supporting the hypothesis that these regions were derived from phage genome including both P-I and P-II consist of DNA sequences with a G+C content of 39.86% and 40.02%, respectively, which differs from the 35.24% G+C content of the core genome. In addition, phage-derived genes including a phage-related lysozyme, head-to-tail joining protein, phage terminase, prophage antirepressor, anti-repressor protein P4 family phage/plasmid primase, and an integrase family protein were identified in both prophage segments. The prophage genes within P-I and P-II do not exhibit colinear arrangement, but are instead mosaics with several hypothetical coding sequences arranged amongst them, suggesting that they are possibly derived from two different prophage integration events (Figure S1). It is unclear if the two phage integration events preceded speciation of ‘*Ca.* L. solanacearum’ and ‘*Ca.* L. asiaticus’ (Figure 3). In addition to the two prophage-like genome sequences, there are a number of prophage-like elements and phage remnants dispersed throughout genome that are presumed to be derived from multiple ancestral bacteriophage integration events (Figure 1 and Figure 3), thus suggesting an involvement of phage integration during gene rearrangement in Liberibacter.

### Carbohydrate uptake, metabolism, and energy metabolism

**Figure 4:**
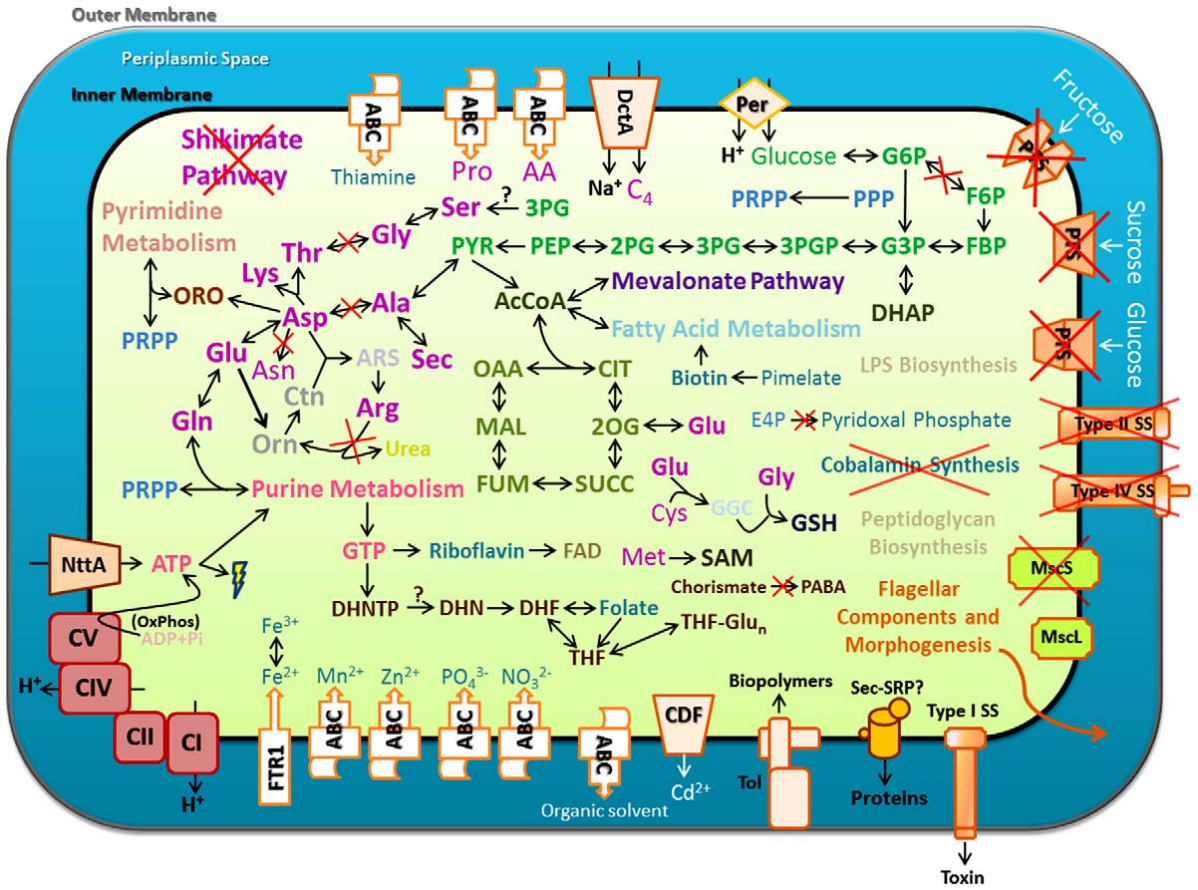
Predicted ‘*Candidatus* Liberibacter solanacearum’ metabolic pathways and general features. Schematic representation of an ‘*Ca.* L. solanacearum’ cell bounded by inner and outer membranes. Cofactors (aqua) and amino acids (violet) that are predicted to be synthesized by ‘*Ca.* L. solanacearum’ are indicated by dark bold text; exogenously-supplied vitamins and amino acids are indicated in faint text. Ions, amino acids, and nucleotides are indicated by common abbreviations. Glucose-6-phosphate (G6P); fructose-6-phosphate (F6P); fructose bisphosphate (FBP); glyceraldehyde-3-phosphate (G3P); 1,3-bisphosphoglycerate (3PGP); 3-phosphoglycerate (3PG); 2-phosphoglycerate (2PG); phosphenolpyruvate (PEP); pyruvate (PYR); orotate (ORO); 5-phosphoribosyl diphosphate (PRPP); ornithine (Orn); citrulline (Ctn); arginosuccinate (ARS); acetyl-CoA (AcCoA); dihydroxyacetone phosphate (DHAP); oxaloacetate (OAA); citrate (CIT); 2-oxoglutarate (2OG); succinate (SUCC); fumarate (FUM); malate (MAL); erythrose-4-phosphate (E4P); S-adenosylmethionine (SAM); gamma-glutamylcysteine (GGC); glutathione (GSH); inorganic phosphate (Pi); flavin adenine dinucleotide (FAD); p-aminobenzoic acid (PABA); dihydroneopterin triphosphate (DHNTP); dihydronepterin (DHN); dihydrofolate (DHF); tetrahydrofolate (THF); polyglutamylated tetrahydrofolate (THF-Glun); dicarboxylate compounds (C4); dicarboxylate transporter (DctA); three-component ABC transporter (ABC);ATP/ADP nucleotide transporter (NttA); glucose permease (Per); phosphotransferase system (PTS); secretion system (SS); cation diffusion facilitator (CDF); high-afinity iron transporter (FTR1); Tol-import pathway (Tol); signal recognition particle secretion pathway (Sec-SRP); complex I (CI); complex II (CII); complex IV (CIV); complex V (CV); small-conductance mechanosensitive ion channel (MscS); large-conductance mechanosensitive ion channel (MscL); oxidative phosphorylation (OxPhos); and pentose phosphate pathway (PPP).

To gain a better understanding of ‘*Ca.* L. solanacearum’ biology, we used the predicted gene inventory of ‘*Ca.* L. solanacearum’ to generate hypotheses about its metabolism and possible lifestyle. As shown in Figure 4, ‘*Ca.* L. solanacearum’ lacks an obvious phosphotransferase system (PTS) [16] for transporting sugars across the inner membrane, but does encode a single glucose/galactose transporter related to the fucose permease family (COG0738) of sugar transporters [17], [18]. Since ‘*Ca.* L. solanacearum’ colonizes phloem tissue of the potato plant it presumably has access to copious amounts of sucrose, fructose, and glucose [19], [20]. However, none of the sequences in its gene repertoire suggests that it is capable of transporting sucrose or fructose across its cell membrane, leading us to hypothesize that glucose is a major form of reduced carbon utilized by ‘*Ca.* L. solanacearum’. Intriguingly, this transporter family is also found in ‘*Ca.* L. asiaticus’ and some *Agrobacterium* species, but is missing from other completely-sequenced *Rhizobiaceae*, suggesting that this transporter may have been lost from some lineages and retained by certain *Agrobacterium* and Liberibacter species.

The ‘*Ca.* L. solanacearum’ genome also encodes a DctA-family dicarboxylate transporter (COG1301) (Figure 4); DctA family members courier a wide range of substrates, including succinate, fumarate, oxaloacetate, and malate [21], [22]. Given that these four compounds, particularly malate, can serve as a primary carbon source to support respiration in root nodule bacteroids [23], it is possible that ‘*Ca.* L. solanacearum’ may also utilize malate as a carbon source when colonizing potato plants, in addition to glucose (above).

‘*Ca.* L. solanacearum’ encodes all the enzymes of the glycolytic pathway, except for glucose-6-phosphate isomerase (EC 5.3.1.9), but could theoretically bypass the early conversions in glycolysis to generate glyceraldehyde-3-phosphate through a partially-complete pentose phosphate pathway (PPP), allowing ‘*Ca.* L. solanacearum’ to produce pyruvate from imported glucose. The ‘*Ca.* L. solanacearum’ genome also encodes all the enzymes required convert pyruvate to acetyl-CoA, which is required for fatty acid metabolism and entry into the TCA cycle. Moreover, ‘*Ca.* L. solanacearum’ possesses all eight subunits needed for functional ATP synthesis (Figure 4), indicating that it can synthesize ATP from ADP and inorganic phosphate similar to other bacteria including ‘*Ca.* L. asiaticus’ (Figure 4) [7]. Not surprisingly, the oxidative phosphorylation pathway of ‘*Ca.* L. solanacearum’ varies only slightly from ‘*Ca.* L. asiaticus’: the HLB bacterium encodes an NADH dehydrogenase (EC 1.6.99.3) which is absent from ‘*Ca.* L. solanacearum’. All other aspects of the oxidative phosphorylation pathways of the two organisms are the same, including previously noted absences of polyphosphate kinase (EC 2.7.4.1), inorganic diphosphatase (EC 3.6.1.1), a cbb3-type cytochrome c oxidase, and the cytochrome bd complex for ‘*Ca.* L. asiaticus’ [7]. In general, these observations lead us to infer that both ‘*Ca.* L. asiaticus’ and ‘*Ca.* L. solanacearum’ seem to have limited capacity for aerobic respiration, consistent with the low-oxygen microenvironments where they are thought to thrive.

As in ‘*Ca.* L. asiaticus’, the ‘*Ca.* L. solanacearum’ genome encodes an ATP/ADP transporter of the NttA family (COG3202) (Figure 4). This transport protein was recently shown to facilitate direct uptake of extracellular ATP and ADP by *E. coli* [8], suggesting that both ‘*Ca.* L. asiaticus’ and ‘*Ca.* L. solanacearum’ can directly import ATP/ADP from extracellular sources. Curiously, orthologs of the NttA transporter family are missing from all other *Rhizobiaceae*, suggesting that this transporter may have been acquired early in the evolution of ‘*Ca.* L. asiaticus’ and ‘*Ca.* L. solanacearum’ through horizontal transfer (Figure S2).

### Amino acid transport and metabolism

‘*Ca.* L. solanacearum’ possesses relatively few of the enzymes required for *de novo* synthesis of amino acids and/or their interconversion (Figure 4). This limited repertoire of biosynthetic genes related to amino acid biosynthesis is consistent with the complement of transporter systems found in ‘*Ca.* L. solanacearum’, as this bacterium encodes at least three complete transporter systems with a cumulative broad range amino acid transport capability: a general L-amino acid ABC transporter system (COG4597, COG0765, COG1126, and COG0834); a proline/glycine-betaine ABC transporter system (COG2113, COG4176, and COG4175); and a DctA-family dicarboxylate (aspartate) transporter (COG1301). Using BLAST analyses, we found that close relatives of all these transporter components occur within the *Rhizobiaceae*, making vertical inheritance a likely source for these putative transporter systems.

**Figure 5:**
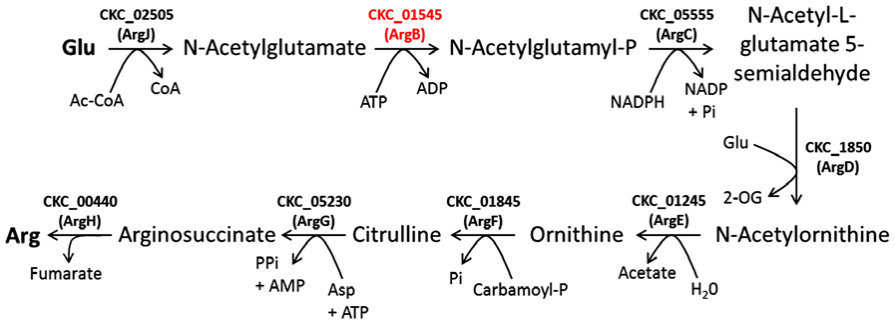
Analysis of the ‘*Candidatus* Liberibacter solanacearum’ arginine biosynthesis pathway. The typical prokaryotic arginine biosynthesis pathway. The NAGK family of enzymes (COG0548) catalyze the second step in arginine biosynthesis and are known as ArgB in many bacteria [106], [107], [108], [109]. In general, NAGKs come in two forms: hexameric arginine-sensitive enzymes and dimeric arginine-insensitive enzymes. The arginine-sensitive varieties of these enzymes typically function as a critical point of feedback inhibition for arginine biosynthesis [24], [110], [111].

Interestingly, comparison of the ‘*Ca.* L. solanacearum’ and ‘*Ca.* L. asiaticus’ genomes revealed one major difference between the two organisms with respect to amino acid metabolism: ‘*Ca.* L. solanacearum’ encodes a full-length N-acetylglutamate kinase (NAGK), while ‘*Ca.* L. asiaticus’ does not [7]. The presence of an NAGK coding sequence in the ‘*Ca.* L. solanacearum’ genome indicates that the ZC bacterium has a complete pathway for the production of arginine from glutamate (Figure 5). The ‘*Ca.* L. solanacearum’ NAGK sequence is highly similar to arginine-sensitive NAGKs and contains all three signatures of an arginine-sensitive NAGK (Figure S3A) [24], indicating that this enzyme probably serves as a point of feedback inhibition for arginine biosynthesis in ‘*Ca.* L. solanacearum’. Phylogenetic analysis of the ‘*Ca.* L. solanacearum’ NAGK sequence places it amongst NAGK sequences of other *Rhizobiaceae* (Figure S3B), indicating this gene was probably inherited vertically from an ancestor of ‘*Ca.* L. solanacearum’. Consistent with this observation, we note that the ‘*Ca.* L. asiaticus’ genome contains a single NAGK-like nucleotide sequence (located between CLIBASIA_01845 and CLIBASIA_01860) that has accumulated several stop codons, suggesting that an ancestor of ‘*Ca.* L. asiaticus’ also encoded a functional NAGK. However, we cannot rule out the possibility of an enzyme with NAGK activity whose sequence is unrelated to the canonical NAGK protein family.

### Nucleotide metabolism

The ‘*Ca.* L. solanacearum’ genome encodes a suite of proteins involved in nucleotide transport and metabolism. The partial pentose phosphate pathway of ‘*Ca.* L. solanacearum’ could provide a supply of 5-phosphoribosyl diphosphate (PRPP) to feed into purine and pyrimidine synthesis (Figure 4). Like ‘*Ca.* L. asiaticus’, ‘*Ca.* L. solanacearum’ probably synthesizes purine nucleotides exclusively through inosine monophosphate and pyrimidine nucleotides exclusively through uridine monophosphate [7]. While we observed no differences between ‘*Ca.* L. asiaticus’ and ‘*Ca.* L. solanacearum’ with regard to nucleotide metabolism, both of these species lack several of the alternative routes of nucleotide synthesis found in closely-related members of the *Rhizobiaceae* family [12], [25], [26], consistent with the highly-specialized lifestyle and reduced genomes of these two disease-associated Liberibacter species.

### Vitamin transport and biosynthesis

In our analysis of the ‘*Ca.* L. solanacearum’ genome, we found only a few genes involved in vitamin uptake. There are no sequences matching complete transporters for riboflavin [27], [28], pyridoxal phosphate [29], niacin [30], cobalamin [29], [31], biotin [32], or folate [33], [34]. This is surprising for a few nutrients, as coding sequences associated with complete biosynthetic pathways for niacin, cobalamin, and pyridoxal phosphate are missing from the ZC-associated bacterium (Figure 4).

While most of the genes involved in thiamine biosynthesis were also absent from the ‘*Ca.* L. solanacearum’ genome, we found all three constituents of a typical prokaryotic thiamine ABC transporter: TbpA (COG4143), ThiP (COG1178), and ThiQ (COG3840) [35]— indicating that ‘*Ca.* L. solanacearum’ derives vitamin B1 exclusively from its environment. The proteins that constitute these transporters in ‘*Ca.* L. solanacearum’ and ‘*Ca.* L. asiaticus’ are more closely related to those of known pathogenic bacteria than to those of the *Rhizobiaceae* (Figure S4).

**Figure 6:**
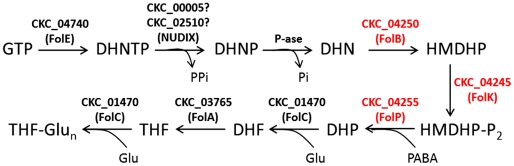
Analysis of the ‘*Candidatus* Liberibacter solanacearum’ folate biosynthetic pathway. A typical prokaryotic folate biosynthetic pathway. Enzymes in red are those encoded by ‘*Ca.* L. solanacearum’ that are not encoded by ‘*Ca.* L. asiaticus’.

In contrast to ‘*Ca.* L. asiaticus’, ‘*Ca.* L. solanacearum’ seems to have a nearly-complete vitamin B9 biosynthesis pathway (Figure 6) capable of performing folate biosynthesis from GTP based on its gene repertoire. ‘*Ca.* L. asiaticus’ lacks *FolB*, *FolK*, and *FolP*-like sequences [7] and probably relies on folate from extracellular sources; these three loci are likely to have originated from a *Rhizobium*-like ancestor (Figure S5). Like many folate-synthesizing bacteria, ‘*Ca.* L. solanacearum’ lacks a FolQ-like pyrophosphatase required to convert 7,8-Dihydroneopterin 3′-triphosphate to dihydroneopterin monophosphate and is also devoid of a PTPS-III bypass enzyme present in select bacteria and protozoans [36], [37]. However, coding sequences for distant relatives the of Nudix-family enzyme involved in this reaction [38] are present in ‘*Ca.* L. solanacearum’ and may provide the pyrophosphatase activity required for the “missing” part of this pathway [37], [39], but we note that neither locus is clustered with *FolP* or *FolC*-like sequences as in some other prokaryotes [40].

### Ion transport and assimilation

**Figure 7:**
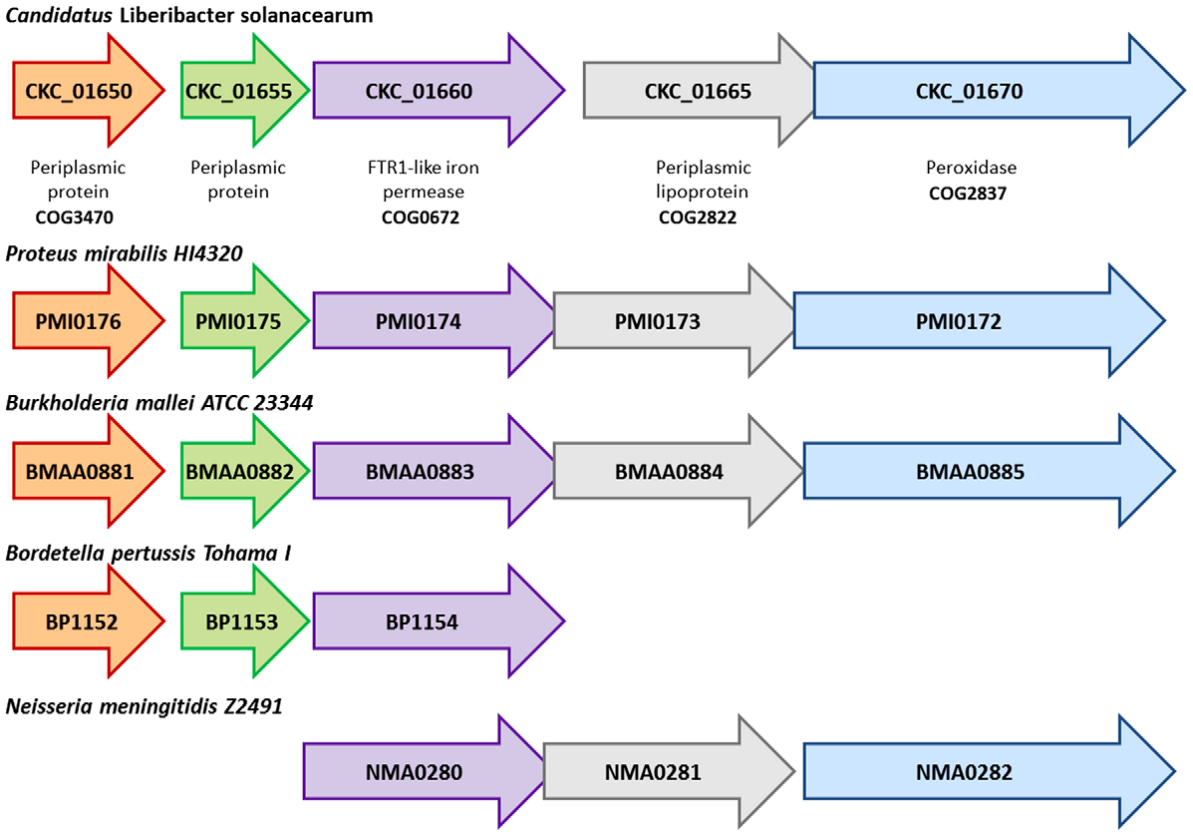
The iron transport and assimilation (*ITA*) gene cluster. Structure of the *ITA* gene cluster from several pathogenic microbes.

Our survey of the ion transporters encoded by the ‘*Ca.* L. solanacearum’ genome revealed multicomponent ABC transporters for phosphate, nitrate, zinc, and manganese (Figure 4). In addition, ‘*Ca.* L. solanacearum’ has also acquired a gene cluster (Figure 7) involved in iron transport and assimilation (*ITA*) that is not present in ‘*Ca.* L. asiaticus’ or any other member of the *Rhizobiaceae*, but is found in several pathogenic genera. The ‘*Ca.* L. solanacearum’ *ITA* gene cluster contains five genes: two predicted periplasmic proteins (CKC_01650 and CKC_01655), an FTR1-like iron permease (CKC_01660), a predicted periplasmic lipoprotein (CKC_01665), and a heme-binding peroxidase (CKC_01670) (Figure 7). The core component of this cluster is *FTR1* (Figure 7); the corresponding ‘*Ca.* L. solanacearum’ protein sequence is closely related to FTR1 sequences from disease-associated *Proteus* and *Providencia* species (Figure S6). Intriguingly, the *ITA* gene cluster is located within a ∼20 kb interval (332644–352525) that contains ∼17 ORFs flanked by two tRNA genes. This interval has a G+C content that is slightly lower (33.77%) than the collective ‘*Ca.* L. solanacearum’ genome (35.07%), but it is unclear if this region is part of a horizontally-acquired genomic island. FTR1-like high-affinity iron transporters have been associated with virulence in several cases and their expression is generally induced in response to iron limitation [41], [42], [43]. As such, it is possible that the *ITA* gene cluster may play a role in causing disease symptoms that resemble iron deficiency in ‘*Ca.* L. solanacearum’-colonized potato plants.

In addition to the high affinity iron transporter, the ‘*Ca.* L. solanacearum’ genome encodes a non-heme ferritin-like protein (CKC_00675, COG1528) [44], [45]. This ferritin-like protein is also found within the ‘*Ca*. L. asiaticus’ genome [7], but absent from the genomes of all other *Rhizobiaceae*. The ferritin superfamily of proteins includes several diverse members that are typically involved in iron storage and detoxification [46], [47], [48]. We hypothesize that this ferritin-like protein may play a critical role in the survival and/or virulence of both ‘*Ca.* L. solanacearum’ and ‘*Ca.* L. asiaticus’, similar to other pathogenic organisms [49], [50], [51], [52]. Curiously, the ‘*Ca.* L. solanacearum’ and ‘*Ca.* L. asiaticus’ ferritin-like sequences are much diverged from other ferritin-like sequences (Figure S7), indicating that the Liberibacter ferritin-like proteins have a unique origin or that extensive isolation of the Liberibacter has led to a novel sequence.

### Sulfur and Nitrogen Assimilation

‘*Ca.* L. solanacearum’, like ‘*Ca.* L. asiaticus’, appears to be incapable of 3-step sulfate reduction and lacks the enzymes required for incorporation of sulfur-containing inorganic compounds into amino acids [7]. While we do not find any evidence for a 3-step sulfate reduction pathway based on the gene prediction, we cannot rule out sulfate reduction through a non-canonical enzymatic process or the use of an alternative terminal electron acceptor under anaerobic conditions.

With regard to nitrogen metabolism, ‘*Ca.* L. solanacearum’ and ‘*Ca.* L. asiaticus’ appear able to incorporate ammonia into glutamine (COG0174), but unlike their nitrogen-fixing relatives both these Liberibacter species have lost the ability to convert nitrogen (N_2_) to ammonia [53], [54]. Strangely, only ‘*Ca.* L. solanacearum’ has retained an ortholog of *Rhizobium sp.* NtrX, a two-component response regulator that has been shown to modulate expression of genes involved in nitrogen fixation [55], [56], [57]. The function of NtrX in the absence of NtrY and *NifA* is unclear, but perhaps NtrX has developed a novel sensor or regulatory function in the lifecycle of the ZC bacterium.

### Cell cycle, growth, and division

The ‘*Ca.* L. solanacearum’ genome encodes orthologs of CtrA, GcrA, and DnaA. These proteins are key regulators of the bacterial cell cycle [58] and may be targets for small-molecule inhibitors aimed at perturbing growth and/or replication of ‘*Ca.* L. solanacearum’. In addition to cell cycle factors, bacterial cell wall synthesis machinery may also be a target for treatment of ZC, based on the efficacy of beta-lactams like penicillin on ‘*Ca.* L. asiaticus’ [59] and considering that the ‘*Ca.* L. asiaticus’ and ‘*Ca.* L. solanacearum’ genomes encode a similar suite of factors involved in peptidoglycan synthesis [60], [61], [62]. Curiously, both ‘*Ca.* L. solanacearum’ and ‘*Ca.* L. asiaticus’ possess only the elongated non-canonical FtsZ (FtsZ2) [63] sequence and lack the shorter FtsZ (FtsZ1) coding sequence found in several members of the *Rhizobiaceae* family [64]. Moreover, only a portion of the genes involved in cell division have been retained in these two Liberibacter species: the *minCDE* gene cluster that helps determine division site placement in bacteria, including the *Rhizobiaceae* [65], [66], has apparently been lost from the Liberibacter lineage. The significance of these gene losses from ‘*Ca.* L. solanacearum’ and ‘*Ca.* L. asiaticus’ is not yet clear.

### DNA replication and repair

Most general bacterial pathways for DNA replication and repair are encoded by the ‘*Ca.* L. solanacearum’ and ‘*Ca.* L. asiaticus’ genomes. However, the ‘*Ca.* L. solanacearum’ genome encodes three known proteins involved in DNA replication and repair that are absent from ‘*Ca.* L. asiaticus’: LexA, DnaE, and RadC. LexA repressors (COG1974) are cleaved in response to UV exposure to activate the bacterial SOS response regulon, which triggers the activity of cellular DNA repair machinery and elicits prophage induction [67], [68]. An ortholog of DnaE (COG0587) is also encoded in the ‘*Ca.* L. solanacearum’ genome. DnaE proteins are involved in lagging strand synthesis in several organisms with low G+C genome content [69]. The expression of this type of polymerase is typically induced as part of the SOS response to facilitate translesion DNA synthesis and typically have high error rates [69], [70]. A RadC ortholog (COG2003) is also encoded in the ‘*Ca.* L. solanacearum’ genome. While not associated with the SOS regulon, its activity is enhanced in response to UV-induced DNA damage [71]. The function of RadC is still unclear, but it is thought to be involved in the repair of DNA strand breaks [71], [72]. We also noted that the RecN DNA repair protein (COG0497) is not encoded by the ‘*Ca.* L. solanacearum’ genome, but is found in the ‘*Ca.* L. asiaticus’ genome [7]. This protein is thought to be involved in the repair of double strand breaks in some bacteria [73], [74].

### Nucleic acid restriction and modification

In our comparison of the ‘*Ca.* L. solanacearum’ and ‘*Ca.* L. asiaticus’ genomes, we identified very few genes of known function (i.e. non-hypothetical) that were unique to the HLB-associated bacterium. Previous work showed that the ‘*Ca.* L. asiaticus’ genome possesses loci encoding complete Type I and Type II restriction-modification systems [7]. The ‘*Ca.* L. solanacearum’ genome encodes a Type I DNA methylase, but lacks any genes coding for restriction enzymes. In general, bacterial DNA restriction-modification systems are thought to be a common defense mechanism against invading phage [75], [76]. These observations are consistent with the presence of at least two large putative phage-integration sites in the ‘*Ca.* L. solanacearum’ genome (Figure 1, Figure 3, and Figure S1).

With further regard to nucleic acid modification, ZC-associated ‘*Ca.* L. solanacearum’ does not encode an ortholog of the tRNA modification enzyme, TrmA. Nearly all organisms, including ‘*Ca.* L. asiaticus’, encode a TrmA-like enzymatic function (EC 2.1.1.35) responsible for the conversion of uridine-54 to ribothymidine during post-transcriptional modification of all tRNAs [77], [78]. TrmA activity is essential for viability in *E. coli* [78] and implicated in stress tolerance in some gram-positive bacteria [79]. The reason for this gene loss from the ZC-associated Liberibacter is not known.

### Cell adherence and motility

Like the HLB-associated Liberibacter, ‘*Ca.* L. solanacearum’ carries a number of genes involved in the assembly of pili and flagella. Pili are involved in cell adhesion in many pathogenic bacteria [80] and ‘*Ca.* L. solanacearum’ appears to encode several tight adherence (Tad) family proteins involved in the assembly surface pili [81]. These genes are located in a ∼8.3 kb region on the ’*Ca.* L. solanacearum’ (138907–147186) and ‘*Ca.* L. asiaticus’ chromosomes (537888–546266), respectively, with both genomes exhibiting a similar gene arrangement within the *Tad* locus.

In contrast to pili, bacterial flagella are generally utilized for bacterial locomotion [82]. These structures have not been observed on the surface of ‘*Ca.* L. asiaticus’ *in planta* [83], though the ‘*Ca.* L. asiaticus’ chromosome does encode for most of the ∼30 factors generally considered to be required for flagellar assembly [7]. Likewise, ‘*Ca.* L. solanacearum’ encodes a nearly-identical set of ∼30 proteins, but preliminary micrographs showing ‘*Ca.* L. solanacearum’ within potato phloem tissue do not clearly resolve flagellar structures [2]. Accordingly, it is not yet known if ‘*Ca.* L. solanacearum’ uses a flagellar apparatus for locomotion inside its host organisms or if the ‘*Ca.* L. solanacearum’ flagellum is assembled only under certain conditions.

### Biomolecular transport pathways associated with virulence

Type I secretion systems (TISSs) are used by many pathogenic bacteria for transport of toxins and other molecules. TISSs are generally composed of a tripartite transporter that forms a contiguous channel through the inner and outer membranes [84], [85]. Evidence for all three of these components was identified in the ‘*Ca.* L. solanacearum’ genome: HlyD (COG0845), PrtD (COG4618), and a distant relative of TolC (COG1538) (Figure 4). Consistent with their function in toxin secretion, the genes encoding orthologs of HlyD and PrtD are clustered together with a gene for an RTX toxin (COG2931) in both the ‘*Ca.* L. solanacearum’ and ‘*Ca.* L. asiaticus’ genomes (Figure S8).

The ‘*Ca.* L. solanacearum’ genome encodes a Tol-like biopolymer transport system (Figure 4), similar to ‘*Ca.* L. asiaticus’ [7] Genes encoding most components of the Sec-SRP [86], [87] transport system were also evident within the ‘*Ca.* L. solanacearum’ genome, though the coding sequence for the SecB chaperone was missing [88], [89]. There was no evidence of a TAT translocation pathway in ‘*Ca.* L. solanacearum’ [90], [91]. Complete Type III and Type IV secretion systems [92], [93] were absent from the ‘*Ca.* L. solanacearum’ genome, similar to the ‘*Ca.* L. asiaticus’ genome [7]. This is not surprising for a pathogen whose route-of-entry into its host probably requires direct injection by an insect vector [94], [95]. ‘*Ca.* L. solanacearum’ is also devoid of Type II secretion pathways and the extracellular oligosaccharide-degrading enzymes they typically courier, consistent with its occupation of the sugar-rich phloem. Conversely, Type II systems are widely used by pathogenic bacteria like *Erwinia*, *Ralstonia*, and *Xanthomonas* species [96], [97], [98] that reside in the plant xylem, where easily-accessible forms of reduced carbon are not readily available.

Our analysis of the complete genome of ‘*Ca.* L. solanacearum’ provides several insights into the physiology of this disease-associated bacterium. More importantly, subsequent comparative analyses with other bacteria revealed key differences between ‘*Ca.* L. solanacearum’ and some of its nearest relatives. We found that, despite having very similar gene content, the organization of the ‘*Ca.* L. solanacearum’ and ‘*Ca.* L. asiaticus’ genomes is quite different (Figure 3), suggesting that several recombination events have helped forge these two genomes since their divergence from a common ancestor. Furthermore, many of these recombination events have likely been mediated by phage infection and integration, based on the presence of several phage-derived gene sequences within both the ‘*Ca.* L. asiaticus’ and ‘*Ca.* L. solanacearum’ genomes (Figure 1, Figure 3, and Figure S1). The absence of genes encoding a complete restriction-modification (RM) system in the ‘*Ca.* L. solanacearum’ genome may make this bacterium highly susceptible to the effects of phage infection and integration. This hypothesis is supported by the presence of two large phage-derived segments within the ‘*Ca.* L. solanacearum’ genome. Although the absence of an RM system may make ‘*Ca.* L. solanacearum’ vulnerable to the effects of prophage integration, it could also lead to an enhanced rate of genome evolution, with ‘*Ca.* L. solanacearum’ acquiring or losing genes through phage-mediated recombination events [99], [100], [101]. It will be interesting to investigate if other strains of ‘*Ca.* L. solanacearum’ lack RM systems as well and to what degree horizontal transfer is currently shaping ‘*Ca.* L. solanacearum’ genomes from different ecosystems.

Based on our comparisons here, it is possible that a few genes and gene clusters have been acquired by ‘*Ca.* L. solanacearum’ and ‘*Ca.* L. asiaticus’ through horizontal transmission. The NttA ATP/ADP transporter present in both ‘*Ca.* L. solanacearum’ and ‘*Ca.* L. asiaticus’ is absent from other *Rhizobiaceae*, but is closely related to the NttA transporter of pathogenic *Rickettsia* (Figure S2). The gene clusters involved in the uptake and sequestration of thiamine and iron are closely related to those found in a variety of pathogenic microbes (Figures S4, Figure 7 and Figure S6) and may play a significant role in the pathogenesis of ZC disease. Moreover, both the *ftr1* and *ftn* loci have a slightly skewed %G+C composition, relative to the core ‘*Ca.* L. solanacearum’ genome. Notably, the FTR1-like sequence identified in these analyses has been associated with varying levels of virulence in other pathogens [102] and may therefore serve as a useful marker for studying populations of ‘*Ca.* L. solanacearum’. Factors such as the NttA and FTR1 transporters could be implicated in disease development by causing energy depletion and nutrient starvation of the host. Functional analyses of these predicted disease-associated factors will likely provide insights into the host-pathogen interactions that occur in ZC and HLB.

While several of the genes highlighted here may be implicated in the development of plant disease symptoms, several of the genes that vary between ‘*Ca.* L. solanacearum’ and ‘*Ca.* L. asiaticus’ appear to be involved in fundamental metabolic pathways. ‘*Ca.* L. solanacearum’ seems to harbor a greater capacity for biosynthesis of amino acids and vitamins compared to ‘*Ca.* L. asiaticus’ (Figure 5 and Figure 6). We conclude that ‘*Ca.* L. solanacearum’ evolved in host environments where arginine and folate are in limited supply, requiring the ZC bacterium to maintain complete biosynthetic pathways for these compounds. In contrast, ‘*Ca.* L. asiaticus’ has lost the capacity to synthesize arginine and folate from glutamate and GTP, respectively— raising the possibility that structural analogs of folate or arginine may be viable treatment options for HLB.

Due to fastidious nature of Liberibacter, the bacterium has not yet been conclusively cultured *in vitro*. Thus, Koch’s postulates have not been fulfilled. Despite of these limitations, the metagenomic approach developed in this study led to the successfully sequencing the entire genome of this bacterium. The assembly from two independent 454 sequencing runs produced a ∼1.26 Mbp circular chromosome which is consistent with the reports from the related species of ‘*Ca.* L. asiaticus’ [7] and ‘*Ca.* L americanus’ [103]. However, some caution should be used as this method fails to identify genetic elements such as plasmids or linear chromosomes. Thus, we need to emphasize that the “missing” or “incomplete” pathways identified in this work are denoted solely on the basis of their absence from the single circular chromosome we were able to assemble from the massive starting pool of sequence data— however, this caveat applies to any metagenomic sequencing effort in which contaminating sequences are present.

Finally, there is a large number of hypothetical proteins encoded by both the ‘*Ca.* L. solanacearum’ and ‘*Ca.* L. asiaticus’ genomes (Table 1). In both cases, greater than 30% of the total coding open reading frames are annotated as encoding hypothetical proteins. This is critical, as several biochemical pathways for key compounds are missing enzymatic activities, and in cases where entire pathways are missing, a known transporter for a particular compound is also absent. While we cannot rule out the presence of additional replicons in ‘*Ca.* L. solanacearum’ or ‘*Ca.* L. asiaticus’ that might encode such functionalities, elucidation of the function of these hypothetical proteins within ‘*Ca.* L. solanacearum’ and ‘*Ca.* L. asiaticus’ will likely provide further fundamental insights into how these organisms survive within their hosts and elicit the disease symptoms associated with ZC and HLB— perhaps leading to the development of several new treatment strategies for these agriculturally and economically-important diseases.

## Materials and Methods

### DNA enrichment and extraction

‘*Ca.* L. solanacearum’ ZC-1 genomic DNA was isolated from potato psyllids (*Bactericera cockerelli* Sulc) collected from potato fields in Dalhart, Texas, USA. Individual psyllids were ground in 50 µL of PBS-BAS buffer (phosphate-buffered saline with 0.1% bovine serum albumin, pH 7.2). A 5 µL aliquot was removed from each psyllid extract sample for DNA isolation. Extracted DNAs were tested for ‘*Ca.* L. solanacearum’ DNA and Ct values were estimated using SYBR real-time PCR with ‘*Ca.* L. solanacearum’- specific primers (Table 1). Psyllid extracts with high titers of ‘*Ca.* L. solanacearum’ DNA (Ct value≤18) were pooled for further enrichment using an immunocapture method [7]. Briefly, the pooled extract was centrifuged at 1,000×*g* for 1 min. The supernatant was transferred to a new tube and centrifuged at 10,000×g for 5 min to collect bacterial cells. Cells were then re-suspended in 500 µL of PBS-BAS buffer. To enrich target bacterial cells, an immunocapture approach was performed using a mixture of rabbit-derived polyclonal antibodies(GenScript Corp, NJ, USA) directed against ‘*Ca.* L. solanacearum’ OMP-A (Ab-OMP-A) and ‘*Ca.* L. solanacearum’ OMP-B (Ab-OMP-B) which were specific for two different synthesized peptides (OMP-A “GKDKKDSYGGKEQLC” and OMP-B “VIRRELGFSEGDPIC”). Rabbit Ab-OMP-A and rabbit Ab-OMP-B were adjusted to 10 µg/mL, respectively. Five microliters of Ab-OMP-A and Ab-OMP-B were added to the cell suspension and gently mixed on a rotator at 40 RPM for 5 hours or overnight at 4°C. Twenty µL of Dynabeads® M-280 Sheep anti-Rabbit IgG (Invitrogen, Carlsbad, CA) were then added to the suspension and incubation was continued for 2 hours. Cells were then collected with a magnetic stand. Prior to DNA extraction, collected cells were re-suspended in 25 µL of DNase solution containing 5 U of DNase I (ABI, Foster City, CA) at 37°C for 30 minutes to help remove residual host DNA. Cells were then collected and washed with 1 mL of PBS-BAS buffer at least 4 times according to the manufacturer's protocol (Invitrogen, Carlsbad, CA). Collected cells were then used for DNA isolation. Precipitated DNA was dissolved in 10 µL of water. One microliter of this DNA preparation was amplified using GenomiPhi whole genome amplification (WGA) kit following the manufacturer's recommendations (GE Life Sciences, NJ, USA). Real-time PCR showed that Ct values of ‘*Ca.* L. solanacearum’ with and without immunocapture were 16.5 and 26, respectively. Thus, immunocapture enriched the target DNA nearly 700 fold (fold = 2^(26–16.5)^).

### PCR confirmation and quantification

SYBR Quantitative real-time PCR was performed with Lso-F forward primers (5′-GTTCCTTTTAAAATTACGTCAGC-3′) and Lso-R reverse primer (5′-GCCGTGTTGTTATATTTTCCG-3′) for ‘*Ca.* L. solanacearum’. A 20 µL of 1× SYBR master mixture (ABI, Foster City, CA) contains 5 µM of forward/reverse primers and 20 ng of genomic DNA obtained either before or after immunocapture-amplified DNA as described above. PCR was carried out using a Bio-Rad IQ5 PCR cycler. With the same amount DNA, samples with the lowest Ct values were selected for WGA. Amplified DNAs were purified by chloroform extraction and ethanol precipitation. DNA was checked on a 1% agarose gel and quantitated using the PicoGreen method (Invitrogen, Carlsbad, CA). DNA was stored at −20°C.

### 454 pyrosequencing

The ‘*Ca.* L. solanacearum’ genome sequence was obtained in two phases. An initial genomic sequence was obtained from a half-plate 454 pyrosequencing run using a Roche GS-FLX Sequencer according to the manufacturer's standard procedures (Roche, Branford, CT, USA). Sequencing data were then assembled using gsAssembler software version 1.1 (Roche, Branford, CT, USA). A second half-plate 454 pyrosequencing run was conducted using a Roche GS-FLX Titanium Series Sequencer located at the University of Iowa's Carver Center for Genomics. Sequences underwent *de novo* assembly with Newbler version 2.0 (Roche, Branford, CT, USA).

### ‘*Candidatus.* Liberibacter solanacearum’ contig confirmation and gap closure

The GenBank accession number for the ‘*Ca.* L. solanacearum’ genome is CP002371. To confirm that the assembled contigs belong to ‘*Ca.* L. solanacearum’, *in silico* analyses were performed using nucleotide BLASTN and BLASTX against the ‘*Ca.* L. asiaticus’ genome (GenBank accession # CP001677) with the cutoff E-value set at 10^−5^. The same analysis was also performed using a number of other phylogenetically-related prokaryotic genomes, including *Rhizobium etli* CIAT 652 (GenBank accession # CP000133), *Agrobacterium tumefaciens* C58 (GenBank accession # AE007869), and the *Wolbachia* endosymbiont of *Culex quinquefasciatus* Pel strain (GenBank accession # AM999887) with cutoff E-value set at 10^−20^. Contigs with top hit to the reference genomes containing 1 kbp or longer were selected for further analysis and PCR primers were designed to anneal to each contig end. PCR confirmation was carried out using DNA extracted from healthy potatoes (negative control) and potatoes exhibiting symptoms of ZC disease. To connect the ‘*Ca.* L. solanacearum’ contigs, the relationships of the contigs were predicted by making an alignment against ‘*Ca.* L. asiaticus’ genome. Primer pairs that bridged two candidate contig ends were amplified using conventional or long-distance PCR protocols. In addition, we also developed two protocols for gap closure of ‘*Ca.* L. solanacearum’ chromosome. The first is an alignment-based contig extension method, conducted by taking 500 bp of both ends of the contig sequence as reference points and performing a BLAST search against all 454 sequence reads. A Perl script was written to extract sequence reads from matching subjects. The extracted sequence reads were then aligned with reference sequences by Cap3 alignment software (http://seq.cs.iastate.edu/). This alignment-based contig extension generated approximately 300–600 bp of consensus sequence that extended beyond each contig end. The second protocol used for gap closure was a genomic walking method [11]. This method usually extends sequences by 1–2 kbp beyond each existing contig end. If extended sequences overlapped with another contig, these connections were confirmed by PCR and then sequenced on an ABI 3130 Genetic Analyzer (ABI, Foster City, CA) to confirm their identity.

### Genome comparison and orthologue identification

Open reading frames within the ‘*Ca.* L. solanacearum’ genome were predicted using Molquest2 version 2.0.4.700 (http://www.molquest.com/). Genome annotation was conducted using multiple reference genomes, including ‘*Ca.* L. asiaticus’ (GenBank accession # CP001677) and other related microbial genome databases obtained from GenBank. Similarity searches were performed by using BLASTX against the nonredundant protein database with a cutoff E value of 10^−20^. Each putative gene was then assigned to a category within the Clusters of Orthologous groups (COG) database. To be consistent with public database annotation, the final complete chromosome sequence was annotated using the NCBI Prokaryotic Genomes Automatic Annotation Pipeline server (PGAAP).

### Molecular phylogenies

Amino acid sequences were retrieved from NCBI databases. In cases where multiple protein sequences were used to infer phylogeny, the amino acid sequences of the proteins of interest were concatenated and then subjected to phylogenetic analysis. The evolutionary history was inferred using the Neighbor-Joining method [104]. The optimal tree is shown in all cases. The percentage of replicate trees in which the associated taxa clustered together in the bootstrap test (1000 replicates) is shown next to the branches. The phylogenetic trees were linearized assuming equal evolutionary rates in all lineages. The trees are drawn to scale, with branch lengths in the same units as those of the evolutionary distances used to infer the phylogenetic trees. The evolutionary distances were computed using the Poisson correction method and are in the units of the number of amino acid substitutions per site. All positions containing gaps and missing data were eliminated from the dataset (Complete deletion option). Phylogenetic analyses were conducted in MEGA4 [105].

## Supporting Information

Figure S1 **Schematic comparison of the ‘** ***Candidatus*** **Liberibacter solanacearum’ P-I and P-II regions.** Alignment of the prophage I (P-I) and prophage II (P-II) sequences in ‘*Ca.* L. solanacearum’ genome. Genes sharing homologous sequence relationships are linked by shading
(TIF)

Figure S2 **Phylogeny of the NttA transporters.** Neighbor-joining tree showing the relationships between NttA protein sequences from several lineages. Bootstrap values are indicated for each node.
(TIF)

Figure S3 **Comparisons of ArgB (NAGK) sequences.** (A) Schematic comparison of ArgB proteins from *E. coli*, ‘*Ca.* L. solanacearum’, and *Pseudomonas aeruginosa*. The N-terminal signature sequence (NTSS), central lysine (K), and C-terminal signature sequences (CTSS) of the arginine-sensitive proteins are indicated. (B) Neighbor-joining tree showing the relationships between ArgB protein sequences from several lineages. Bootstrap values are indicated for each node.
(TIF)

Figure S4 **Phylogenetic analysis of the ‘** ***Candidatus*** **Liberibacter solanacearum’ thiamine transport system.** Neighbor-joining tree showing the relationships between concatenated sequences for all three hypothesized thiamine transporter components for the lineages shown. Bootstrap values are indicated for each node.
(TIF)

Figure S5 **Phylogenetic analysis of the folate biosynthesis components FolB-FolK-FolP from ‘** ***Candidatus*** **Liberibacter solanacearum’.** Neighbor-joining tree showing the relationships between concatenated sequences for three folate synthesis proteins (FolB-FolK-FolP) for the lineages shown. Bootstrap values are indicated for each node.
(TIF)

Figure S6 **Phylogenetic analysis of FTR1 family members.** Neighbor-joining tree showing the relationships between FTR1 protein sequences from the lineages shown. Bootstrap values are indicated for each node.
(TIF)

Figure S7 **Phylogenetic analysis of the ferritin-like proteins.** Neighbor-joining tree showing the relationships between ferritin (Ftn) protein sequences from several lineages. Bootstrap values are indicated for each node.
(TIF)

Figure S8 **The ‘** ***Candidatus*** **Liberibacter asiaticus’ and ‘** ***Candidatus*** **Liberibacter solanacearum’ RTX toxin transport loci.** Schematics of the loci encoding components of the ‘*Ca.* L. asiaticus’ and ‘*Ca.* L. solanacearum’ Type I secretion system.
(TIF)

Table S1 **Conventional and long-distance PCR primers used for ‘** ***Candidatus*** **Liberibacter. solanacearum’ sequence confirmation and gap close in this study.**
(XLSX)

Table S2 **Primers used for genomic walking.**
(XLSX)

Table S3 **List of ‘** ***Candidatus*** **Liberibacter solanacearum’and ‘** ***Candidatus*** **Liberibacter asiaticus’ best hits.**
(XLSX)

Table S4 **List of ‘** ***Candidatus*** **Liberibacter solanacearum’-specific and ‘** ***Candidatus*** **Liberibacter asiaticus’-specific CDS.**
(XLSX)

Table S5 **Summary of transporter analysis from ‘** ***Candidatus*** **Liberibacter solanacearum’.**
(XLSX)
