# Supplementary figures and images for: The Complete Genome Sequence of ‘Candidatus Liberibacter solanacearum’, the Bacterium Associated with Potato Zebra Chip Disease
Source: PLoS One. 2011 Apr 28;6(4):e19135. doi: 10.1371/journal.pone.0019135 (PMC3084294; doi:10.1371/journal.pone.0019135)

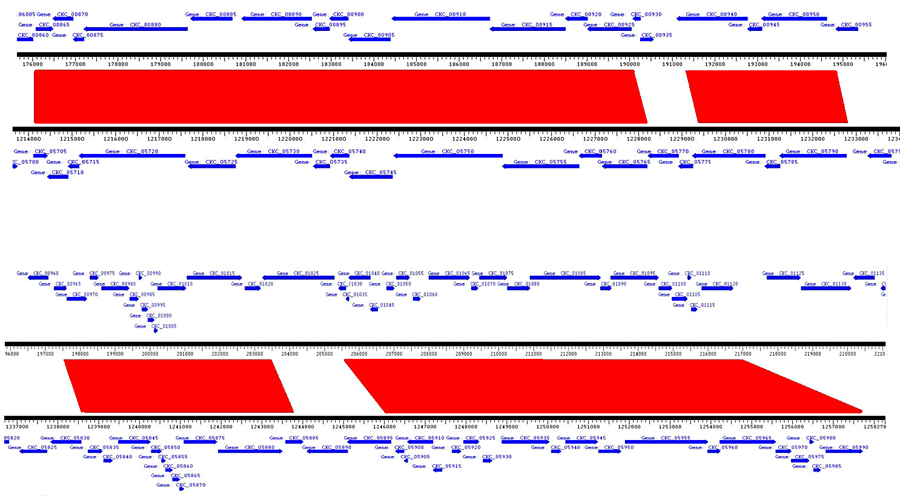

Supplement: Figure S1 — Schematic comparison of the ‘ Candidatus Liberibacter solanacearum’ P-I and P-II regions. Alignment of the prophage I (P-I) and prophage II (P-II) sequences in ‘Ca. L. solanacearum’ genome. Genes sharing homologous sequence relationships are linked by shading (TIF) [file pone.0019135.s001.tif]

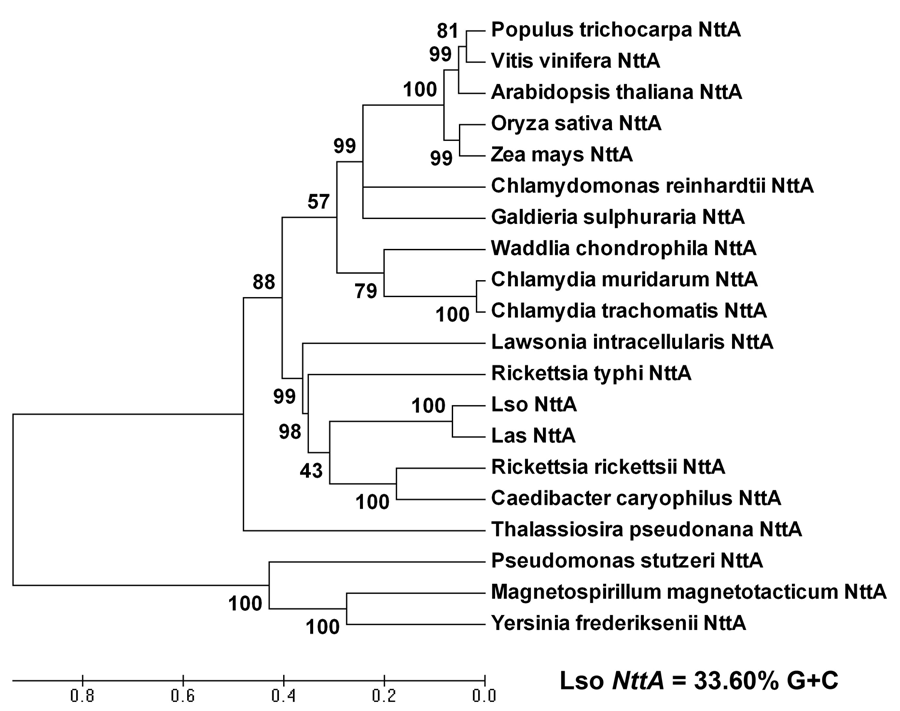

Supplement: Figure S2 — Phylogeny of the NttA transporters. Neighbor-joining tree showing the relationships between NttA protein sequences from several lineages. Bootstrap values are indicated for each node. (TIF) [file pone.0019135.s002.tif]

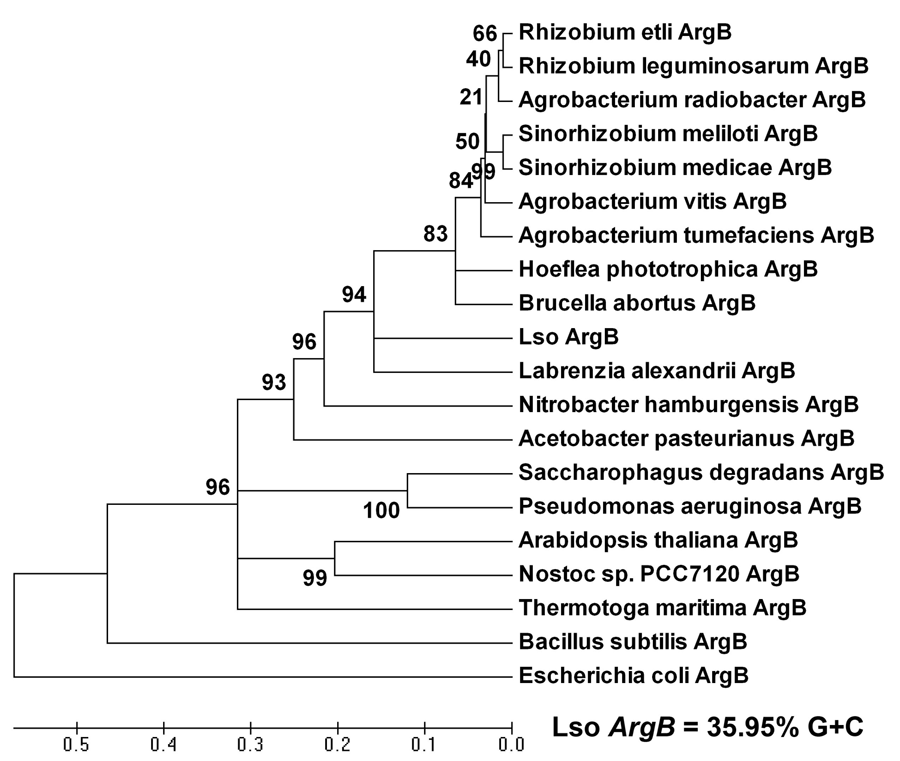

Supplement: Figure S3 — Comparisons of ArgB (NAGK) sequences. (A) Schematic comparison of ArgB proteins from E. coli, ‘Ca. L. solanacearum’, and Pseudomonas aeruginosa. The N-terminal signature sequence (NTSS), central lysine (K), and C-terminal signature sequences (CTSS) of the arginine-sensitive proteins are indicated. (B) Neighbor-joining tree showing the relationships between ArgB protein sequences from several lineages. Bootstrap values are indicated for each node. (TIF) [file pone.0019135.s003.tif]

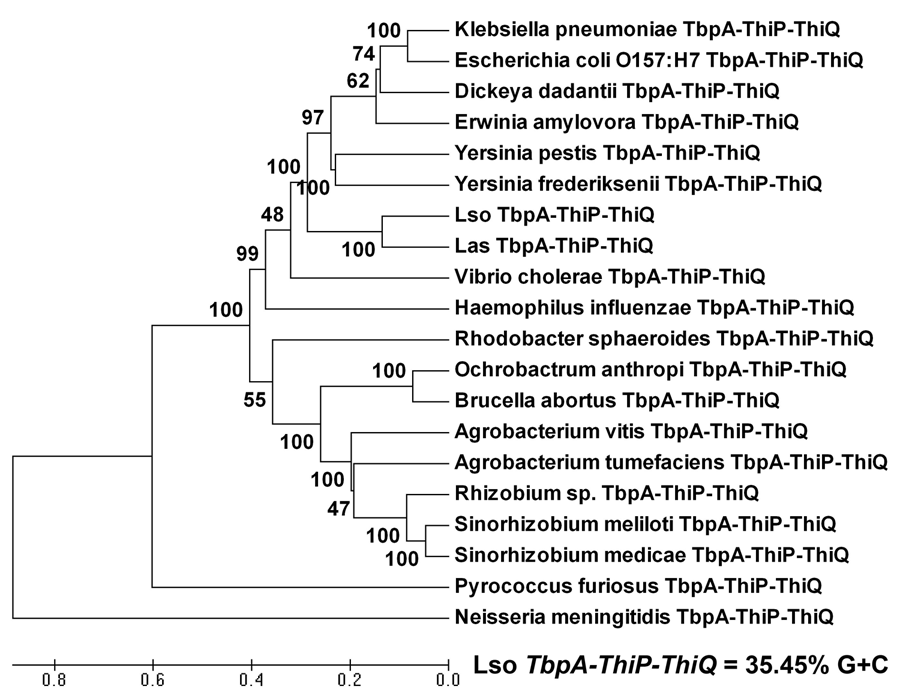

Supplement: Figure S4 — Phylogenetic analysis of the ‘ Candidatus Liberibacter solanacearum’ thiamine transport system. Neighbor-joining tree showing the relationships between concatenated sequences for all three hypothesized thiamine transporter components for the lineages shown. Bootstrap values are indicated for each node. (TIF) [file pone.0019135.s004.tif]

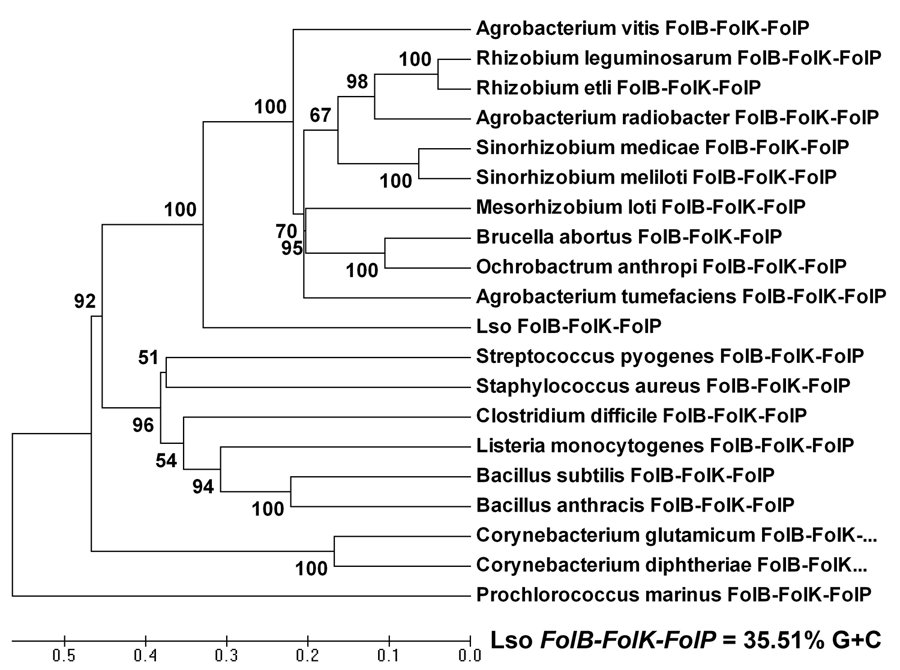

Supplement: Figure S5 — Phylogenetic analysis of the folate biosynthesis components FolB-FolK-FolP from ‘ Candidatus Liberibacter solanacearum’. Neighbor-joining tree showing the relationships between concatenated sequences for three folate synthesis proteins (FolB-FolK-FolP) for the lineages shown. Bootstrap values are indicated for each node. (TIF) [file pone.0019135.s005.tif]

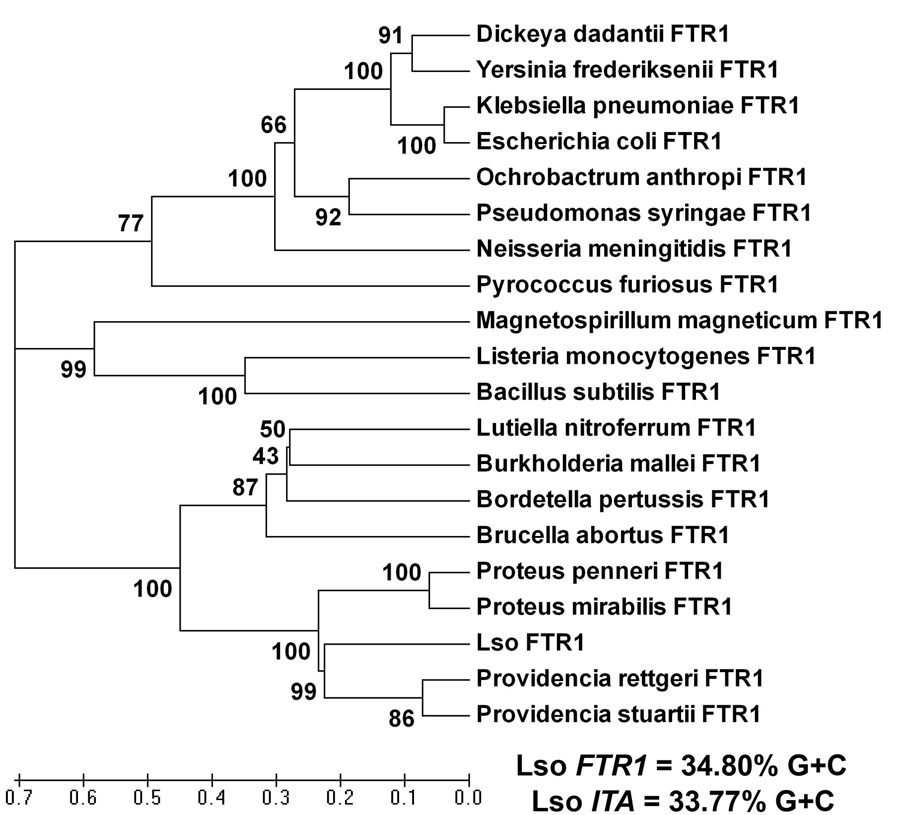

Supplement: Figure S6 — Phylogenetic analysis of FTR1 family members. Neighbor-joining tree showing the relationships between FTR1 protein sequences from the lineages shown. Bootstrap values are indicated for each node. (TIF) [file pone.0019135.s006.tif]

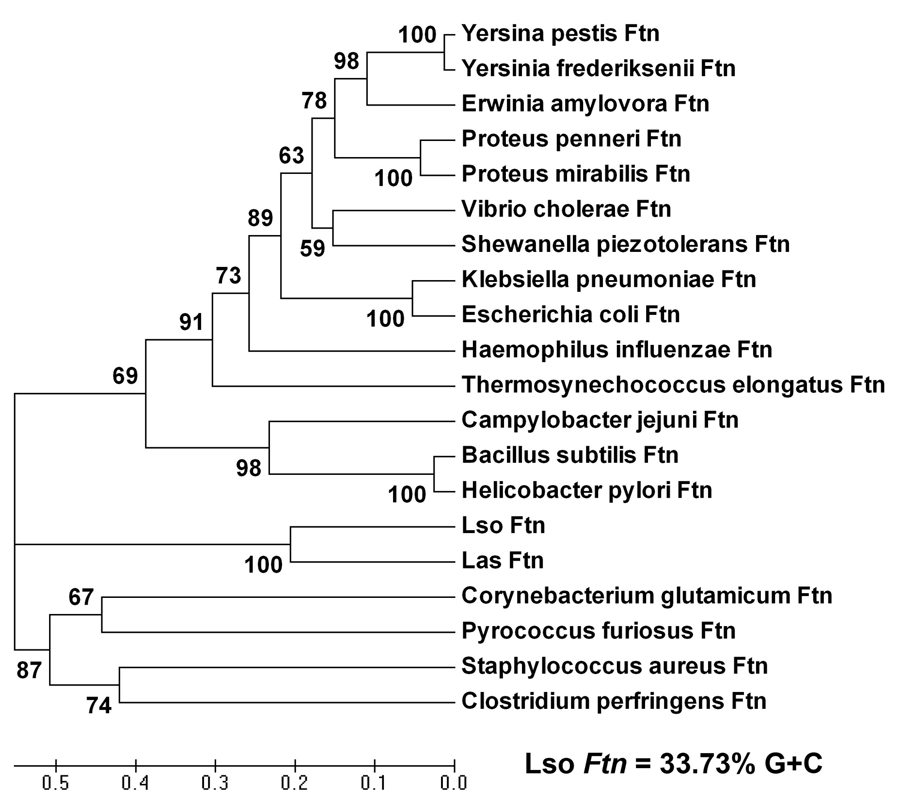

Supplement: Figure S7 — Phylogenetic analysis of the ferritin-like proteins. Neighbor-joining tree showing the relationships between ferritin (Ftn) protein sequences from several lineages. Bootstrap values are indicated for each node. (TIF) [file pone.0019135.s007.tif]

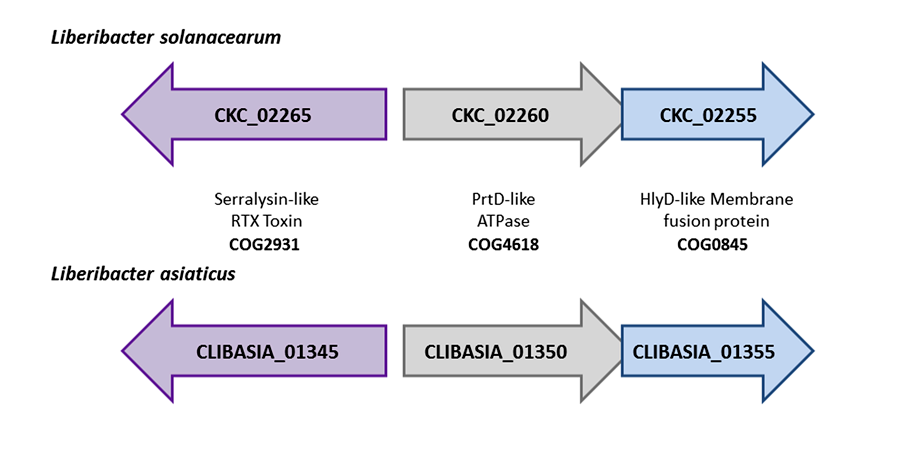

Supplement: Figure S8 — The ‘ Candidatus Liberibacter asiaticus’ and ‘ Candidatus Liberibacter solanacearum’ RTX toxin transport loci. Schematics of the loci encoding components of the ‘Ca. L. asiaticus’ and ‘Ca. L. solanacearum’ Type I secretion system. (TIF) [file pone.0019135.s008.tif]
